# Suicide Trends Among Persons Aged 10–24 Years — United States, 1994–2012

**Published:** 2015-03-06

**Authors:** Erin M. Sullivan, Joseph L. Annest, Thomas R Simon, Feijun Luo, Linda L. Dahlberg

**Affiliations:** 1 Division of Analysis, Research, and Practice Integration; National Center for Injury Prevention and Control, CDC; 2 Division of Violence Prevention, National Center for Injury Prevention and Control, CDC

Suicide is the second leading cause of death among persons aged 10–24 years in the United States and accounted for 5,178 deaths in this age group in 2012 (1). Firearm, suffocation (including hanging), and poisoning (including drug overdose) are the three most common mechanisms of suicide in the United States. Previous reports have noted that trends in suicide rates vary by mechanism and by age group in the United States (2), with increasing rates of suffocation suicides among young persons (3–5). To test whether this increase is continuing and to determine whether it varies by demographic subgroups among persons aged 10–24 years, CDC analyzed National Vital Statistics System mortality data for the period 1994–2012. Trends in suicide rates were examined by sex, age group, race/ethnicity, region of residence, and mechanism of suicide. Results of the analysis indicated that, during 1994–2012, suicide rates by suffocation increased, on average, by 6.7% and 2.2% annually for females and males, respectively. Increases in suffocation suicide rates occurred across demographic and geographic subgroups during this period. Clinicians, hotline staff and others who work with young persons need to be aware of current trends in suffocation suicides in this group so that they can accurately assess risk and educate families. Media coverage of suicide incidents and clusters should follow established guidelines to avoid exacerbating risk for “suicide contagion” among vulnerable young persons.* Suicide contagion is a process by which exposure to the suicide or suicidal behavior of one or more persons influences others who are already vulnerable and thinking about suicide to attempt or die by suicide. Early prevention strategies are needed to reduce the likelihood of young persons developing suicidal thoughts and behavior.

CDC’s Web-Based Injury Statistics Query and Reporting System was used to compile National Vital Statistics System data on annual suicide counts and rates for persons aged 10–24 years from 1994 (when the suicide rate peaked in this age group) to 2012. Transition of cause of death coding from the ninth to the 10th revision of the *International Classification of Diseases* (ICD) in 1999 had minimal effect on examining trends in suicide rates during the study period because the comparability ratio of ICD-10 to ICD-9 is very close to 1 (0.9962).† Suicide rates per 100,000 were calculated using bridged-race population estimates from the U.S. Census Bureau; age-adjusted rates were computed using the United States standard 2000 population. Trends in suicide rates were examined for all mechanisms combined, by sex, by the three leading mechanisms of suicide (firearm, suffocation, and poisoning), and by all other mechanisms combined for each of three 5-year age groups (10–14, 15–19, and 20–24 years), sex, race/ethnicity, and U.S. Census region. Trend analysis of poisoning suicide rates among some subcategories was limited because of unstable rates resulting from small death counts.

Joinpoint regression was used to test the significance of trends and to calculate the annual percent change (APC) during 1994–2012. For trend analyses of suicide rates among persons aged 10–24 years during that period, joinpoint regressions were performed for each leading mechanism of suicide across the selected demographic/geographic subgroups. Average annual percent change (AAPC) was calculated and used to facilitate comparison of trends across groups with different numbers of joinpoints. AAPC (reported as a single statistic for each subgroup) takes a weighted average of the annual percent change calculated across joinpoints. For comparison of overall suicide rate by sex, both AAPC and APC (using >0 joinpoints) were calculated. Rate ratios (RRs) with 95% confidence intervals (CIs) were calculated to compare suffocation suicide rates for 2012 with those for 1994, by sex, age group, race/ethnicity, and U.S. Census region.

Overall age-adjusted suicide rates by sex fluctuated somewhat during 1994–2012, but rates among males were consistently much higher than among females. In 1994 rates were 15.7 per 100,000 among males compared with 2.7 among females. In 2012, rates were 11.9 among males compared with 3.2 among females. Among males, age-adjusted suicide rates decreased significantly from 1994 to 2007 (APC [1994–1999] = −5.7, p<0.001; APC [1999–2007] = −1.2, p<0.001), and increased significantly from 2007 to 2012 (APC = 2.4, p<0.001). Among females, age-adjusted suicide rates decreased significantly during 1994–2001 (APC = −4.4, p<0.001), increased (but not significantly) during 2001–2004 (APC = 6.9, p=0.058), decreased (but not significantly) during 2004–2007 (APC = −2.9, p=0.378) and increased significantly during 2007–2012 (APC = 6.9, p<0.001). AAPCs over the study period for males and females were −1.5 (p<0.001) and 0.7 (p=0.385), respectively.

Among males aged 10–24 years, firearm was the leading mechanism of suicide, whereas, among females, suffocation surpassed firearm in 2001 as the leading mechanism (Figure). Suicide rate trends by mechanism were similar for males and females over the study period. In general, firearm suicide rates decreased and suffocation suicide rates increased, while rates for suicide by poisoning decreased slightly and rates for suicide by all other mechanisms combined remained relatively unchanged.

For both males and females, age-adjusted firearm suicide rates decreased significantly from 1994 to 2012 (males: from 10.9 to 5.9 per 100,000; AAPC = −3.4, p<0.001; females: from 1.5 to 0.8; AAPC= −3.6, p=0.002) with a notable decline from 1994 to 2007 followed by an uptick from 2007 to 2012 (APC = 2.4, p<0.001 for males; APC = 5.6, p<0.001 for females). During 1994–2012, downward trends were significant across all four age groups, all racial/ethnic groups, and all U.S. Census regions. The largest significant decreases in firearm suicide rates during 1994–2012 were among persons aged 15–19 years (AAPC = −4.2, p<0.001), Asian/Pacific Islanders (AAPC = −6.9, p<0.001), and persons living in the West (AAPC = −4.4, p<0.001).

Poisoning suicide rates were considerably lower than either firearm suicide or suffocation suicide rates. From 1994 to 2012, poisoning suicide rates decreased significantly (from 1.0 per 100,000 to 0.6; AAPC = −2.3, p<0.001) among males and decreased among females (but not significantly) (from 0.6 per 100,000 to 0.4; AAPC = −1.8, p=0.078). Trend analysis of poisoning suicide rates among some subgroups was limited because of unstable rates resulting from small death counts.

What is already known on this topic?

Among persons aged 10–24 years, suicide rates are higher in males than in females. Suicide rates by suffocation (including hanging) have been increasing among females in this age group since the early 1990s.

What is added by this report?

Overall age-adjusted suicide rates among persons aged 10–24 years in 1994 were 15.7 per 100,000 among males compared with 2.7 among females. In 2012, these rates were 11.9 per 100,000 among males and 3.2 among females. During 1994–2012, age-adjusted suffocation suicide rates continued to increase among females aged 10–24 years and also increased significantly, although less sharply, among males in this age group. These rates have increased across all racial/ethnic groups and U.S. Census regions.

What are the implications for public health practice?

These results highlight the increased use of suffocation as a method of suicide among young persons. Professionals who work with young persons and their families need to be aware of the trend in this highly lethal method when asking about suicide plans and when working to reduce suicide risk. These results also underscore the importance of early prevention of suicidal behavior and effective intervention for youth and young adults at greater risk for suicide.

For both males and females, age-adjusted suffocation rates increased significantly from 1994 to 2012 (males: from 3.0 to 4.5 per 100,000 population; AAPC = 2.2, p<0.001; females: from 0.5 to 1.7; AAPC = 6.7, p<0.001) (Table). Suffocation suicide rates increased among all age groups, races/ethnicities, and regions, and the AAPCs all were significant. The largest increases in suffocation suicide rates were among persons aged 15–19 years (AAPC = 3.3, p<0.001), American Indians/Alaska Natives (AAPC = 4.9, p<0.001), and persons living in the Midwest (AAPC = 3.9, p<0.001). Rate ratios were highest for females (3.6), persons aged 15–19 years (1.9), non-Hispanic whites (1.9), and persons living in the Midwest (2.1) (Table).

## Discussion

Increases in suffocation suicide rates, reported in earlier studies (3–5), continued through 2012 among females and males aged 10–24 years across all races/ethnicities and U.S. Census regions. Since the early 1980s, firearm had been the most common mechanism of suicide among those aged 10–24 years (1). However, suffocation surpassed firearm as the most common mechanism of suicide among females in 2001. An uptick in firearm suicide rates was observed for males and females after 2007. Increases in suffocation suicide rates also have been reported in older age groups, especially middle-aged adults (2,3). These trends are concerning because suffocation as a suicide mechanism has a high lethality rate, typically 69%–84% (3). By comparison, lethality rates for firearms and poisoning in 2010 were 81% and 2%, respectively (3). Additional research (e.g., perceptions about hanging as a method of suicide) is needed to understand why suffocation suicide rates are increasing (6).

The findings in this report are subject to at least three limitations. First the findings are subject to variation among state coroner/medical examiners regarding determination of manner of death, especially for poisoning, as recorded on the death certificate (7). Second, suicide rates likely are an underestimate of the actual prevalence because suicides might be undercounted in the National Vital Statistics System (7). Finally, suicide rates might be affected by death certificate race/ethnicity misclassification, particularly for American Indians/Alaska Natives.§

The increased use and high lethality of suffocation as a suicide method underscores the importance of early prevention strategies to reduce onset of suicidal thoughts in young persons and to help identify persons who are contemplating suicide or who are at greater risk for suicide (8). National data indicate that 17% of high school students reported seriously considering suicide and 8% reported making one or more suicide attempts in the preceding 12 months (9). Clinicians, hotline workers, and other practitioners who are trained to assess suicide plans and to intervene with young persons should be aware of the increased use and high lethality of suffocation as a suicide method. The National Strategy for Suicide Prevention encourages a comprehensive approach to suicide prevention that includes activities for enhancing social support, problem-solving skills, and other protective factors to prevent suicidal behavior; increasing training in recognizing risk factors and making appropriate referrals; expanding access to social services; reducing stigma and other barriers to seeking help; and providing responsible media reporting to reduce contagion and to enhance awareness that suicide is preventable (10). Media coverage that provides details about suicide methods has the potential to increase contagion among vulnerable youth. Established recommendations for reporting on suicide are designed to reduce contagion, provide hope, and raise awareness about warning signs and actions that readers can take to help those close to them. The National Strategy for Suicide Prevention calls for integration of suicide prevention into a range of programs and services because strategies that promote overall health and build positive relationships are critically important for reducing suicidal thoughts, attempts, and deaths.

## Figures and Tables

**FIGURE:**
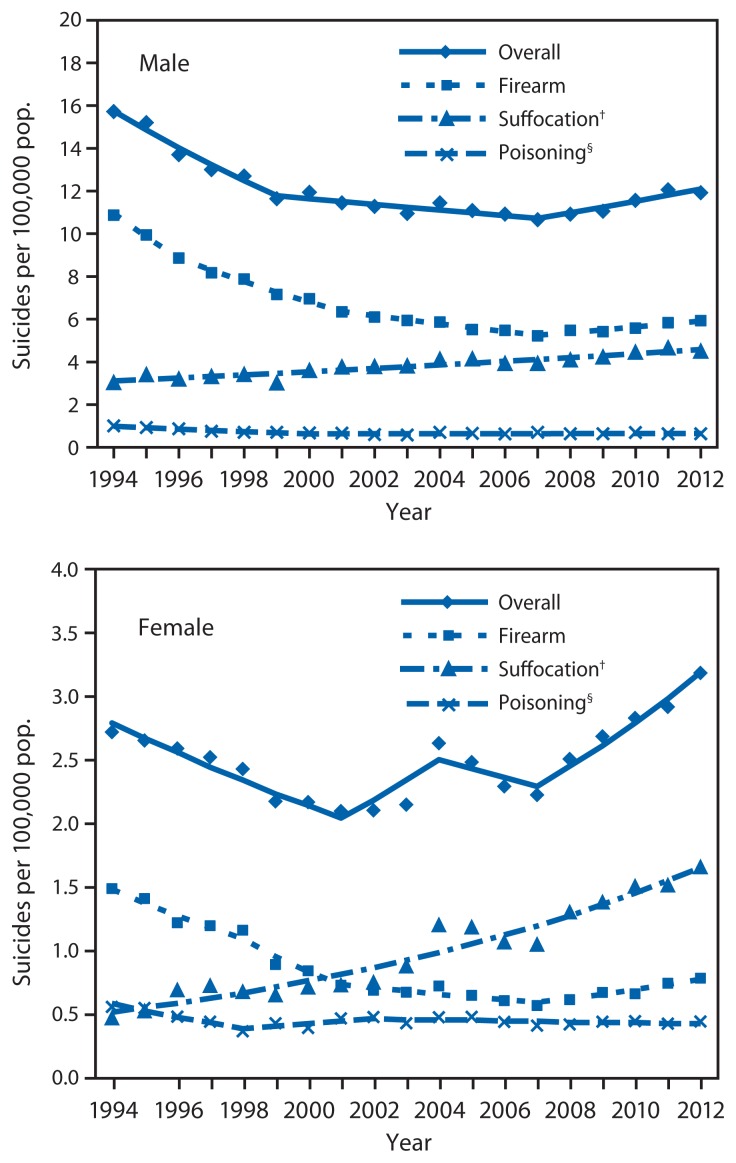
Age-adjusted suicide rates among persons aged 10–24 years, by sex and mechanism — United States, 1994–2012^*^ ^*^ Symbols (diamond, square, triangle, x) representing joinpoints are displayed on the line graphs because, for both males and females, some of the suicide rates were best fitted by multiple segments of lines (number of joinpoints >0). ^†^ Including hanging. ^§^ Including drug overdose.

**TABLE:** Numbers and age-adjusted rates per 100,000 population of suicides by suffocation* among persons aged 10–24 years, by selected characteristics — United States, 1994 and 2012

Characteristic	1994		2012		Annual % change†	p-value	Rate ratio	(95% CI)
	
	No.	Rate	No.	Rate				
**Overall**	**997**	**1.8**	**2,077**	**3.1**	**3.0**	**<0.001**	**1.8**	**(1.6–1.9)**
**Sex**								
Male	871	3.0	1,545	4.5	2.2	<0.001	1.5	(1.4–1.6)
Female	126	0.5	532	1.7	6.7	<0.001	3.6	(3.0–4.4)
**Age group (yrs)**								
10–14	103	0.5	195	0.9	1.5	0.018	1.7	(1.4–2.2)
15–19	344	1.9	787	3.7	3.3	<0.001	1.9	(1.7–2.2)
20–24	550	3.0	1,095	4.9	2.5§	<0.001	1.7	(1.5–1.8)
**Race/Ethnicity**								
White, non-Hispanic	676	1.8	1,326	3.5	3.3	<0.001	1.9	(1.7–2.1)
Black, non-Hispanic	109	1.4	216	2.1	2.9	<0.001	1.6	(1.2–2.0)
Hispanic	110	1.5	360	2.6	2.9	<0.001	1.8	(1.4–2.2)
Asian/PI, non-Hispanic	40	1.8	88	2.4	1.4§	<0.001	1.3	(0.9–2.0)
AI/AN, non-Hispanic	40	8.9	82	12.2	4.9	<0.001	1.4	(0.9–2.0)
**U.S. Census region** ¶								
Northeast	186	1.8	323	2.8	2.2	<0.001	1.6	(1.3–1.9)
South	312	1.6	704	2.8	3.0§	0.002	1.8	(1.6–2.1)
Midwest	239	1.8	535	3.8	3.9	<0.001	2.1	(1.8–2.4)
West	260	2.1	515	3.2	2.7	<0.001	1.5	(1.3–1.8)

**Abbreviations:** CI = confidence interval; PI = Pacific Islander; AI/AN = American Indian/Alaska Native.

* Includes hanging.

† Computed using joinpoint regression of annual suffocation suicide rates during 1994–2012.

§ Regression analyses indicated that models with more than zero joinpoints provided the best fit to the data for these three subgroups. For consistency, the average annual percentage change was computed as a weighted average of the annual percentage changes for each model, allowing for comparison across groups with differing numbers of joinpoints.

¶ *Northeast:* Connecticut, Maine, Massachusetts, New Hampshire, New Jersey, New York, Pennsylvania, Rhode Island, Vermont; *Midwest:* Illinois, Indiana, Iowa, Kansas, Michigan, Minnesota, Missouri, Nebraska, North Dakota, Ohio, South Dakota, Wisconsin; *South:* Alabama, Arkansas, Delaware, District of Columbia, Florida, Georgia, Kentucky, Louisiana, Maryland, Mississippi, North Carolina, Oklahoma, South Carolina, Tennessee, Texas, Virginia, West Virginia; *West*: Alaska, Arizona, California, Colorado, Hawaii, Idaho, Montana, Nevada, New Mexico, Oregon, Utah, Washington, Wyoming.

## Footnotes

* Available at http://reportingonsuicide.org/Recommendations2012.pdf.

† Additional information available at http://www.cdc.gov/nchs/data/nvsr/nvsr49/nvsr49_02.pdf.

§ Additional information available at http://wonder.cdc.gov/wonder/help/cmf/sr02_148.pdf.
